# Tackling unresolved questions in forest ecology: The past and future role of simulation models

**DOI:** 10.1002/ece3.7391

**Published:** 2021-03-30

**Authors:** Isabelle Maréchaux, Fanny Langerwisch, Andreas Huth, Harald Bugmann, Xavier Morin, Christopher P.O. Reyer, Rupert Seidl, Alessio Collalti, Mateus Dantas de Paula, Rico Fischer, Martin Gutsch, Manfred J. Lexer, Heike Lischke, Anja Rammig, Edna Rödig, Boris Sakschewski, Franziska Taubert, Kirsten Thonicke, Giorgio Vacchiano, Friedrich J. Bohn

**Affiliations:** ^1^ INRAE CIRAD CNRS AMAP Univ Montpellier Montpellier France; ^2^ Department of Ecology and Environmental Sciences Palacký University Olomouc Olomouc Czech Republic; ^3^ Department of Water Resources and Environmental Modeling Czech University of Life Sciences Prague Czech Republic; ^4^ Helmholtz Centre for Environmental Research ‐ UFZ Leipzig Germany; ^5^ German Centre for Integrative Biodiversity Research (iDiv) Halle‐Jena‐Leipzig Leipzig Germany; ^6^ Institute of Environmental Systems Research Osnabrück University Osnabrück Germany; ^7^ Forest Ecology Institute of Terrestrial Ecosystems ETH Zürich Zurich Switzerland; ^8^ EPHE CEFE CNRS Univ Montpellier Univ Paul Valéry Montpellier IRD Montpellier France; ^9^ Potsdam Institute for Climate Impact Research (PIK) Member of the Leibniz Association Potsdam Germany; ^10^ Institute of Silviculture University of Natural Resources and Life Sciences (BOKU) Vienna Austria; ^11^ TUM School of Life Sciences Technical University of Munich Freising Germany; ^12^ Forest Modelling Lab Institute for Agriculture and Forestry Systems in the Mediterranean National Research Council of Italy (CNR‐ISAFOM) Perugia (PG) Italy; ^13^ Department of Innovation in Biological, Agro‐food and Forest Systems University of Tuscia Viterbo Italy; ^14^ SBiK‐F ‐ Senckenberg Biodiversity and Climate Research Centre Frankfurt am Main Germany; ^15^ University of Natural Resources and Life Sciences Vienna Austria; ^16^ Dynamic Macroecology Land Change Science Swiss Federal Institute for Forest, Snow and Landscape Research WSL Birmensdorf Switzerland; ^17^ DISAA Università degli Studi di Milano Milano Italy

## Abstract

1. Understanding the processes that shape forest functioning, structure, and diversity remains challenging, although data on forest systems are being collected at a rapid pace and across scales. Forest models have a long history in bridging data with ecological knowledge and can simulate forest dynamics over spatio‐temporal scales unreachable by most empirical investigations.
2. We describe the development that different forest modelling communities have followed to underpin the leverage that simulation models offer for advancing our understanding of forest ecosystems.
3. Using three widely applied but contrasting approaches – species distribution models, individual‐based forest models, and dynamic global vegetation models – as examples, we show how scientific and technical advances have led models to transgress their initial objectives and limitations. We provide an overview of recent model applications on current important ecological topics and pinpoint ten key questions that could, and should, be tackled with forest models in the next decade.
4. Synthesis. This overview shows that forest models, due to their complementarity and mutual enrichment, represent an invaluable toolkit to address a wide range of fundamental and applied ecological questions, hence fostering a deeper understanding of forest dynamics in the context of global change.

## Graphical abstract

Forest models can help understanding the processes that shape forest functioning, structure and diversity, since they can can simulate forest dynamics over spatio‐temporal scales unreachable by most empirical investigations. Here we describe the development of three widely applied but contrasting forest mo−delling approaches — species distribution models, individual‐based models and dynamic global vegetation models. We provide an overview of recent model applications and pinpoint ten key questions that could, and should, be tackled with forest models in the next decade.

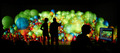

## 1 UNRESOLVED QUESTIONS IN FOREST ECOLOGY: CHALLENGES AND WAYS FORWARD

Forests cover about 30% of the Earth's land surface, store almost half of the terrestrial carbon, are pivotal for the global carbon balance, supply important resources to billions of people, and host more than half of Earth's known biodiversity (Jenkins et al., 2013; Pan et al., 2011; Ramage et al., 2017; Vira et al., 2015). Yet, ongoing and future environmental changes put forests at risk. This raises the demand for a more detailed understanding of forest dynamics and for assessing the future of forest ecosystems to continuously update our knowledge base and provide information to decision‐makers (IPBES, 2016; Mori, 2017; Mouquet et al., 2015; United Nations, 2014). Forest ecology is, however, confronted with the challenge of investigating complex systems that are characterized by long‐term dynamics over large spatial scales, and therefore many questions remain unresolved (Sutherland et al., 2013).

In the context of global biodiversity loss, for instance, understanding the link between forest biodiversity and ecosystem functioning is of high interest (Naeem et al., 2009). However, long‐term effects remain underexplored and underlying mechanisms are still under debate (Loreau et al., 2001; Scherer‐Lorenzen, 2014). Similarly, forest responses to perturbations can be complex and non‐linear, as they involve multiple processes operating at various scales, from canopy physiology across demography to long‐term adaptation and compositional changes. As a result, forest dynamics remain difficult to forecast (Felton & Smith, 2017; Ives & Carpenter, 2007), but understanding the underlying processes is critical in an epoch of global change, including changes in the intensity and frequency of climate extremes and disturbances (Field et al., 2012; Reichstein et al., 2013; Seidl et al., 2017). As another illustration, quantifying forest carbon stocks and fluxes and identifying their drivers are important tasks, in particular to inform climate change mitigation policies such as REDD (Reducing Emissions from Deforestation and Degradation; Gibbs et al., 2007). However, substantial uncertainties remain in estimated carbon and other element stocks and fluxes associated with forests locally and worldwide (Bonan, 2008; Pan et al., 2011; Ploton, Mortier, Réjou‐Méchain, et al., 2020; Réjou‐Méchain et al., 2019).

Knowledge gaps may result from the lack of theoretical frameworks (Courchamp et al., 2015; Franklin et al., 2020) and/or from the limited availability of suitable data, which are often costly and time‐consuming to collect. As trees are typically long‐lived, experiments and field monitoring should extend over multiple decades to capture meaningful trends, which is a temporal coverage still out of reach of most empirical studies and prevents their repeatability (Schnitzer & Carson, 2016). Although an increasing amount of field and remote‐sensing data have been made available at various spatial and temporal extent and resolution over the past decades, their integration into a coherent picture remains a considerable challenge (Chave, 2013; Estes et al., 2018; Levin, 1992).

In parallel, a variety of vegetation and forest models have been continuously developed by different scientific communities and for different purposes. Orchestrating the interplay of various data and theories with forest modelling has been identified as a promising approach to tackle current research challenges (Franklin et al., 2020; Shugart et al., 2015; van der Sande et al., 2017; Zuidema et al., 2013). While fundamentally relying on the basic knowledge developed through theoretical considerations or empirical studies, models themselves represent an efficient tool to, under given assumptions, generate virtual data or perform virtual experiments out of reach of empirical investigations in terms of temporal and spatial scope as well as number of replicates (e.g., Fyllas et al., 2017; Morin et al., 2018; Schmitt et al., 2019). For example, using a forest dynamics model, Bohn and Huth (2017) created a database of 500,000 virtual forest plots varying in forest composition and structure, allowing to explore the drivers of the temperature sensitivity of productivity in temperate forests.

In addition, providing anticipatory predictions of possible futures, models can be used to test hypotheses about processes (explanatory or corroboratory predictions; Maris et al., 2018; Mouquet et al. 2015) by applying a range of scenarios or comparing different ways to model processes, for example, between model versions or different models, and confronting them with data (e.g., Collalti et al., 2019; Fisher et al., 2006; Fleischer et al., 2019; Langan et al., 2017; Lovenduski & Bonan, 2017; Morin et al., 2021; Sakschewski et al., 2016). For example, using different versions of the same forest model, Collalti, Tjoelker, et al., (2020) tested two ecological theories about plant respiration. Models can thus also prove useful to pinpoint data and knowledge gaps and hence further guide the design of new experiments and empirical studies (Medlyn et al., 2016; Norby et al., 2016; Rykiel, 1996; Van Nes & Scheffer, 2005).

In the following, we evidence how the availability of various forest modelling approaches and decades of experience in assimilating observational knowledge into models offer invaluable tools to address key fundamental and applied ecological questions on forests. To do so, we first present three widely used but contrasting modelling approaches to simulate forests, namely, species distribution models (SDMs), individual‐based forest models (IBMs), and dynamic global vegetation models (DGVMs). Our aim is to illustrate the diversity and complementarity of forest modelling approaches. We then show how recent developments have allowed models to tackle similar questions, transgressing their own historical objectives and limitations, and paving the way to new synergies and opportunities for forest ecology. Finally, we sketch out how forest models, singly and in combination, could take on an increasing role in addressing a variety of key ecological questions in the future.

## 2 DIFFERENT APPROACHES TO MODEL FORESTS

Different approaches have been developed to model forest ecosystems and community dynamics, as well as forest cover and tree species distributions. They range from basic theoretical models such as neutral models (Hubbell, 2001), through models of growth patterns of individual trees, to forest stand or landscape models (Shifley et al., 2017), or global vegetation models (Prentice et al., 2007). Depending on the specific objectives of the scientists, the model representations of vegetation biodiversity, structure, or biogeochemical processes have various degrees of complexity due to different degrees of aggregation or abstraction resulting from the differing assumptions used to construct the respective model.

The three model types we briefly present here — SDMs, IBMs, and DGVMs — cover a gradient from models that initially focused on a detailed representation of individual species to models that gave initial emphasis to the representation of forest structure and tree demography, to others that focused on the representation of biogeochemical processes. We chose these widely used model types to illustrate the variety of modelling approaches that can be and have been used to address forest ecology questions in the context of global change. In the following, we present these three approaches by ordering them along a gradient of decreasing resolution of biodiversity representation and increasing resolution of biogeochemical process representation, acknowledging that other forms of presentation could alternatively have been used.

### 2.1 Species distribution models

Species distribution models (Booth et al., 2014; Guisan et al., 2017) focus on the spatial distribution of species and how it varies with environmental drivers. SDMs have their origin in flora distribution maps, which laid the concepts of biogeography (Grisebach, 1872; Humboldt, 1849). The development and increased usage of SDMs across a wide array of taxa and environments have relied on several technical advances (Elith & Leathwick, 2009; Guisan & Thuiller, 2005), namely, statistical approaches (e.g., *MaxEnt*), methods for physical environment mapping (e.g., remote‐sensing techniques), and increasingly coordinated efforts to compile knowledge on species distributions. All these approaches have been boosted by geographic information systems (GIS).

Species distribution models rely on the concept of the ecological niche (Guisan & Thuiller, 2005; Hutchinson, 1957; Soberón, 2007) and can be described as a two‐step process. First, the ecological niche representation of a species is built in environmental space, based on known records in places where environmental conditions have been described. Second, each geographic location is assigned a probability of occurrence for the species, based on the niche model (Elith & Leathwick, 2009).

Species distribution models thus require little information about the processes from which species distributions result. This can be an advantage, for example, for poorly known taxa in demand of conservation actions. Also, by looking for a best model fit in species niche modelling, important environmental drivers of spatial species patterns may be revealed (Bertrand et al., 2012; Thuiller et al., 2003). SDMs have also been used to predict species distributions under future environmental conditions, such as species invasion or climate change (Thuiller, 2003; Thuiller et al., 2005). However, key assumptions of SDMs, mainly that species are at equilibrium with their environment (Václavík & Meentemeyer, 2012), that species can always migrate to suitable environments, and that the species–environment relationships are valid beyond the range of model calibration, may be violated under such applications (Araújo & Pearson, 2005; Svenning & Skov, 2004; Veloz et al., 2012). Classical SDMs are further limited to a species‐by‐species approach and, thus, typically overlook the role of species interactions in shaping species distributions (Dormann et al., 2018), although more recent developments aim at including species interactions (e.g., Meier et al. 2011). Additionally, the spatial autocorrelation (SAC) inherent in both species distribution and environmental variables can bias the estimated performance of SDMs (Bahn & McGill, 2007; Fourcade et al., 2018; Journé et al., 2020), calling for care when using extrapolations from SDMs (Sofaer et al., 2018). However, at the same time, accounting for SAC in SDMs by various methods (Dormann et al., 2007; Václavík et al., 2012) can improve their accuracy because SAC is often a result of important ecological processes (e.g., dispersal limitation, colonization time lag) that drive species distributions.

The integration of eco‐physiological and demographic processes into SDMs is likely critical to inferring species distributions in novel environments or under no‐present analogue conditions (Dormann et al., 2012; Kearney & Porter, 2009; Urban et al., 2016). Models that combine the traditional approach of SDMs with process‐based information (Morin & Lechowicz, 2008; Thuiller et al., 2008), such as dispersal limitation or phenology, have been developed (Bykova et al., 2012; Chuine & Beaubien, 2001; Duputié et al., 2015; Kleidon & Mooney, 2000; Nobis & Normand, 2014; Stephenson, 1990). Progress has also been made to integrate species competition as biotic factors influencing species realized niche (Leathwick & Austin, 2001; Meier et al., 2011) and further extend these ideas to full ecological communities (Ferrier & Guisan, 2006).

### 2.2 Individual‐based forest models

There is a long tradition in ecology and forestry to use individual‐based models to answer a broad range of scientific questions. This type of models simulates the development of each individual tree within a forest stand. A key component is the interaction between single trees (e.g., by shading), which is crucial for tree growth and influences community dynamics. The simulation of individual trees allows for capturing not only forest structure but also tree species diversity. A widely known type of IBMs are forest gap models (Bugmann, 2001; Huston et al., 1988; Shugart, 1984). As first developed for forest stands in North America, they have since become one of the most widely used model types in ecology (Botkin et al., 1972; Shugart et al., 2018; Shugart & West, 1977).

In the gap model approach, a forest stand is described as a mosaic of forest patches. The dynamics of the forest at the stand scale emerge from the growth, mortality, establishment, and competition of individual trees (Bugmann, 2001; Porté & Bartelink, 2002). The vertical distribution of leaves is used to calculate the light availability for each tree, which affects growth and mortality. Competition with neighboring trees usually happens within a predefined competition range, where all trees compete for resources such as light, water, and nutrients. Due to the individual‐based concept, these models are able to describe different aspects of successional dynamics (mosaic dynamics, e.g., Watt, 1947) and natural heterogeneity of forest stands (Knapp et al., 2018). The coupling of biogeochemical processes is modelled in an aggregated way in forest gap models, using the concept of limiting factors (affecting tree growth rates). Gap models can simulate the impact of temperature, precipitation, CO_2_, and light on tree dynamics, and thus on forest productivity, biomass, and species composition (Overpeck, Rind, & Goldberg, 1990; Pastor & Post, 1988; Solomon, 1986). Some early studies already included the carbon and nutrient cycles (Pastor & Post, 1986). Gap models are typically used with annual time steps for the demographic processes of growth, recruitment, and mortality, with finer embedded timestep to update the simulated environment.

Modules for forest management (Huth & Ditzer, 2001; Liu & Ashton, 1995; Mina et al., 2017) and disturbances like fire (Fischer, 2013; Kercher & Axelrod, 1984), browsing (Didion et al., 2009; Seagle & Liang, 2001), or windthrow (Seidl et al., 2011, 2014) have been included in subsequent studies. Tree mortality can thus be described as an exogenous process (e.g., by disturbances), but also as a growth‐dependent and/or intrinsic process (e.g., Keane et al., 2001). Although gap models were first developed for temperate forests in the USA, they were soon applied also for European temperate (Bugmann, 1996; Kienast, 1987) and boreal forests (Leemans & Prentice, 1989). Forest gap models have also been developed for tropical forests (Bossel & Krieger, 1991; Doyle, 1981; Fischer et al., 2016; Köhler & Huth, 1998). To simplify the high species richness of these forests, tropical gap models typically simulate forest succession by grouping tree species that share similar ecological features into plant functional types (PFTs). The gap model approach was also extended beyond forests, for example, to grassland systems (Coffin & Lauenroth, 1990; Schmid et al., 2021; Taubert et al., 2020).

From the 1990s onwards, models that keep track of the positions of each tree in a finer‐grained grid (i.e., they are spatially explicit) and thus allow for a more detailed computation of tree light availability have been developed (Chave, 1999; Maréchaux & Chave, 2017; Pacala et al., 1996; Pretzsch et al., 2002). Other developments have led to a more explicit representation of processes, for example by including a more detailed temperature and CO_2_ dependence of photosynthesis and respiration, or more detailed water and carbon cycles or site fertility (Fischer et al., 2016; Maréchaux & Chave, 2017). Similarly, by taking advantage of comprehensive trait databases or long‐term inventories and the detailed information they provide on tree life histories, novel parameterizations have allowed for simulating hundreds of species within diverse forest communities (Maréchaux & Chave, 2017; Rüger et al., 2019). Other stand‐based models were designed to describe forest stand structure dynamics driven by eco‐physiological processes in higher detail and at finer time scales (Kramer et al., 2002; Medlyn et al., 2007; Morales et al., 2005), although often at the cost of lower temporal or spatial coverage. IBMs have since been used to address a wide variety of basic and applied research questions, concerning for example forest development under climate change, assessments of management scenarios, or the drivers of tree community composition (Bohn et al., 2014; Bugmann & Pfister, 2000; Fischer et al., 2016; Seidl et al., 2012; Shugart et al., 2018). Modern extensions of these models also allow for simulations of forests at large spatial scales (i.e., from forest landscapes to entire countries or continents; Rödig et al., 2017; Sato et al., 2007; Scherstjanoi et al., 2014; Thom et al., 2017; Xiaodong & Shugart, 2005).

### 2.3 Dynamic global vegetation models

Dynamic global vegetation models have their origin in four research areas: plant geography, biogeochemistry, vegetation dynamics, and biophysics (Prentice et al., 2007), with IBIS, HYBRID, and LPJ being among the first DGVMs (Cramer et al., 2001). DGVMs have initially been developed to represent the interaction between vegetation and the global carbon cycle as stand‐alone models, but also to represent vegetation dynamics in the context of Earth System Models, that is, along with models of the atmosphere (General Circulation Models), the oceans, and the cryosphere.

Dynamic global vegetation models simulate vegetation dynamics from half‐hourly to monthly time steps at the global scale, driven by climate, atmospheric CO_2_ concentration, and soil information, using plant physiology and biogeochemistry to explain biogeography (Krinner et al., 2005; Sitch et al., 2003). This approach results in the prediction of the large‐scale distribution of potential natural vegetation. The main components of DGVMs are representations of photosynthesis, respiration, leaf transpiration, carbon allocation, mortality, and disturbance. The exchange of carbon and water fluxes is represented at the leaf level by stomatal conductance (Ball et al., 1987; Collatz et al., 1991; Rogers et al., 2017).

Describing vegetation dynamics at the global scale inevitably entails strong model simplifications to represent vegetation. Therefore, DGVMs use PFTs to aggregate functionally similar species to represent functional properties at the biome scale. Usually global vegetation is described with between 5 and 14 PFTs by differentiating life form, leaf form, phenology, or photosynthetic pathway, for example, tropical broad‐leaved raingreen tree or C_3_ grasses (Prentice et al., 2007; Woodward & Cramer, 1996). Hence, these PFTs represent a less detailed description of species diversity within forest communities than the ones used in IBMs. Additionally, DGVMs often are used to conduct simulations using a relatively coarse‐grained grid (typically of 0.5° lat/lon resolution) in which the characteristics within each cell are assumed to be spatially homogenous, simulating average individuals per PFT, where several of them can compete within one grid cell. Hence, local competition processes are simplified and the influence of spatial structure within this coarse grid cell is neglected. Moreover, DGVMs typically apply the ‘big‐leaf’ approach, whereby photosynthesis of the PFTs is simulated based on one photosynthetic surface throughout the grid cell. Most stand‐alone DGVMs are not initialized with any observed vegetation distribution, nor with measured data for carbon and water pools. The global PFT and carbon pool distribution is instead determined by the given abiotic conditions and PFT‐specific characteristics, that is, in so‐called “spin‐up” simulations. Hence, each change in abiotic conditions (e.g., climate change) results in a reaction of the vegetation.

Although DGVMs were originally developed to simulate potential natural vegetation, including fire disturbance (Lenihan et al., 1998; Thonicke et al., 2001), they have been advanced by simulating land‐use (Bondeau et al., 2007; Boysen et al., 2016; Langerwisch et al., 2017; Rolinski et al., 2018), water management (Jägermeyr et al., 2015), and forest management (Bellassen et al., 2010). In order to account for the role of nutrient deposition in vegetation dynamics and its interaction with the global carbon cycle, several DGVMs have been further developed to include an explicit representation of the nitrogen and phosphorus cycle (von Bloh et al., 2018; Goll et al., 2017; Reed et al., 2015; Smith et al., 2014; Wang et al., 2010). Similarly, a more explicit representation of tree hydraulics and water flows has been developed in some DGVMs to better assess the effect of climatic change on evapotranspiration and drought‐related mortality (Bonan et al., 2014; Hickler et al., 2006; Langan et al., 2017). The need for a more realistic representation of vegetation structure and biodiversity to improve the predictive power of DGVMs has been highlighted as an important pathway to improving their predictive power (McMahon et al., 2011; Quillet et al., 2010). To achieve this, several developments have been made to include a finer representation of vegetation demographic processes (Fisher et al., 2018; Hickler et al., 2012; Moorcroft et al., 2001; Smith et al., 2001) and functional diversity (Pavlick et al., 2013; Sakschewski et al., 2015; Scheiter et al., 2013; Verheijen et al., 2015). Lately, also seed dispersal of trees and therefore the ability for tree species migration has been implemented into hybrid DGVMs, which represent a combination of a forest gap model with a DGVM (Lehsten et al., 2019; Snell & Cowling, 2015).

We will henceforth use the terms “forest models” and “forest modelling” to describe the variety of models that have been used to simulate forest systems, among which the three model types described above are widely used examples, acknowledging that each model type is also used to simulate other ecological systems.

## 3 FROM MODEL COMPLEMENTARITY TO SYNERGIES: OPPORTUNITIES FOR FOREST ECOLOGY

### 3.1 Converging trajectories of model developments

As illustrated above, the different forest modelling approaches were initially motivated by different specific objectives, leading to different choices and simplifications in the representation of actual vegetation (Table 1). DGVMs originally focused on bio‐geochemical processes as the exchange of carbon and water between vegetation and atmosphere at the global scale, at the cost of a realistic representation of forest diversity, competition, and structure. Conversely, SDMs adopted a species‐level representation of vegetation diversity, but have long relied on a correlative‐only approach, bypassing the mechanistic processes underlying species distribution. Similarly, IBMs typically used a finer‐grained representation of vegetation structure than DGVMs, as they simulate many individuals, and focus on the competition among them, but often at the cost of a coarser representation of some processes such as gas exchange or water flow, typically using empirical equations fitted at a coarser temporal resolution than DGVMs.

**TABLE 1:** Advantages, limitations, and challenges of three different approaches to model forests: species distribution models (SDMs), individual‐based models (IBMs), and dynamic global vegetation models (DGVMs)

	SDMs	IBMs	DGVMs
Advantages	allow a quick assessment of potential climate‐change vulnerability can serve as a coarse filter for more detailed/process‐based approaches are easily applicable to many taxa due to low data and computational demands, as well as available R‐packages and methods	simulate the growth and demography of every tree in a forest from decades to centuries can easily integrate field data since forest monitoring is mainly done at the tree level are able to simulate dynamics of forest structure and project changes in species composition by including important ecological processes (e.g., competition between species) can integrate disturbances, climate change and forest management are mostly process‐based and therefore useful for extrapolations to new conditions	simulate vegetation on large spatial (up to global) and temporal scales (decades to centuries) simulate climate impacts on vegetation dynamics and associated biogeochemical and water cycles due to the process‐based simulation of stocks and fluxes are able to consider physiological and plant‐competition processes and increasingly plant‐trait diversity can incorporate managed grasslands and crop growth under land‐use change.
Limitations	represent potential rather than realized species niches are static models, as equilibrium with environment is assumed, which can lead to misinterpretations by stakeholders their accuracy depends strongly on the spatial resolution, as there can be strong effects of spatial autocorrelation.	are data demanding for parameterization and initialization can be computationally demanding to apply at large spatial scales (countries, continents) since millions of trees have to be simulated in these cases can raise problem of overfitting and erroneous extrapolations when calibrated using local field data	often represent species diversity using plant functional types (PFTs), and species‐specific parameterization is limited by a lack of information for important parameters (e.g., on ecophysiology). have high computational demand at large scales have often a poor representation of forest structure and certain ecological processes (e.g., seed dispersal, forest regrowth, tree mortality) show – by design – no or only rudimentary simulation of forest management
Challenges and future developments	deal with missing absence data include more ecological processes and species interactions include genetic variability include demographic processes and dispersal limitations better account for the impact of extreme climatic events	speed up the parameterization step upscale while keeping essential behavior improve the coupling of remote‐sensing data with model outputs include intra‐specific variation and plasticity more realistically represent below‐ground processes	increase the number of PFTs to an optimal number, to represent major competing functional groups simulate actual vegetation consisting of managed forests and remaining natural vegetation as monitored by forest inventories or remote sensing improve the implementation of vegetation structure to allow its integration at the global scale improve the representation of ecological processes (e.g., vegetation re‐growth and seed dispersal)

However, multiple scientific and technical advances (see Box 1, Table 2) have allowed for overcoming the constraints that modelers initially were facing. Each of these model types has thus been gaining in efficiency and capabilities as illustrated by the aforementioned recent model developments: next‐generation DGVMs strive to explicitly represent tree demography and diversity within PFTs, and account for forest structure, IBMs refine their representation of biogeochemical cycles, while SDMs endeavor to include process‐based information. In doing so, their trajectories of development have been progressively converging. As a result, each model type has broadened its field of applications beyond its initial scope, creating synergies among models, including model coupling, to address key ecological research questions in a mutually informative way.

**TABLE 2:** Types of available forest data

Short description	Extent (space; time)	Resolution (space; time)	Examples (references and links)
Data type: experimental data			
Monitoring of plant responses to a set of controlled or manipulated biotic (e.g., competition) or abiotic (e.g., nutrients, climate) conditions	Very local‐to‐stand‐scale; variable	Small‐scale; variable	Bussotti et al., (2018); FACE experiment (Free‐Air CO2 enrichments), Norby et al., (2016); rainfall manipulation, Grossiord et al., (2018); Meir et al., (2015)
Data type: tree performance data			
Direct or indirect measurements of components of tree performance or functioning, such as tree growth (e.g., tree‐ring analysis, automatic dendrometers), resource use, (e.g., sapflow), or reproduction (e.g., seed traps)	Local; from snapshots to tree life span.	individual to forest stand; Intra‐annual to annual	tree‐ring databases, Treydte et al., (2007), https://www.ncdc.noaa.gov/data‐access/paleoclimatology‐data/datasets/tree‐ring; tree sapflow database, Poyatos et al., (2016); seed production database, Ascoli et al., (2017); Muller‐Landau et al., (2008)
Data type: trait data			
Measurement of plant individual features (morphological, physiological or phenological) which impacts components of individual performance	Local; snapshots or repeated over e.g., season, ontogeny	Individual to species; punctual or repeated over e.g., season, ontogeny.	TRY, Kattge et al., (2011); sPlot, Bruelheide et al., (2019)
Data type: species presence records			
Report of presence or absence of species in localities	Across species range, from local to global; snapshots or repeated over longer term	Variable; punctual	Global Biodiversity Information Facilities, GBIF, https://www.gbif.org/
Data type: inventory data			
Systematic identification and size measurements of all trees above a given size threshold within a forest stand	Local stands or stand network; snapshots or repeated over longer term	Individual; punctual or typically from seasonal to every few years.	German national inventory, https://bwi.info/start.aspx?lang=eng; CTFS‐ForestGeo, https://forestgeo.si.edu/
Data type: eddy‐flux data			
Measurement of vertical turbulent fluxes of water and CO_2_ between the atmosphere and the vegetation layer	Stand (tower footprint of typically few hectares); continuous measurements over years	Stand; Half‐hourly	FLUXNET, Baldocchi et al., (2001); Pastorello et al., (2020)
Data type: remote‐sensing observations			
Record of vegetation characteristics and abiotic conditions from above, based on propagated signal such as electromagnetic waves, either active (e.g., LiDAR, RADAR) or passive (visible light).	Regional, global; covering several years	Spaceborne: down to meter‐scale; several measurements per year.	e.g., MODIS: https://modis.ornl.gov/cgi‐bin/MODIS/global/subset.pl; Justice et al., (2002); Running et al., (2004)
	Stand to regional scale; snapshot or repeated over e.g., seasons or years.	Airborne: down to cm‐scale; Punctual or repeated flights	https://gliht.gsfc.nasa.gov/; Goetz and Dubayah, (2011); Zolkos et al., (2013)
	Local to stand scale; snapshots or repeated over e.g., weeks or seasons	Drone‐based: down to cm‐scale; punctual or repeated flights	Brede et al., (2017); Park et al., (2019); Roşca et al., (2018)
	Local to stand scale; snapshots or repeated over e.g., seasons or years	Terrestrial: down to mm‐scale; mostly punctual	Disney, (2018); Takoudjou et al., (2018)

**Box 1**: New levers to foster model development for forest ecology

Forest model development and predictive ability have been constrained by different factors. Forest models are data‐demanding across the different steps of model development and application, from a robust parameterization of the multiple processes related to plant life cycle and physiology for diverse plant types, species or individuals, to the initialization and validation of forest simulations over large spatial and temporal scales. Fortunately, data availability is increasing at a high pace (Table 2). Global plant trait databases (e.g., TRY, Kattge et al., 2011) gather data of commonly measured traits (e.g., leaf mass per area or wood density) for a wide range of species, and this effort is being expanded to other traits (e.g., stem and leaf drought tolerance, Bartlett et al., 2012; Choat et al., 2012; fine root traits, Iversen et al., 2017; litter decomposition rates, Brovkin et al., 2012). This fosters a systematic model trait‐based parameterization for a range of plant species and individuals. If data coverage remains incomplete however (Kattge et al., 2020), the combination of organization principles such as natural selection‐based optimality or entropy maximization to constraint plant and ecosystem behavior can alleviate the data demand for parameterization while improving model predictive ability (Franklin et al., 2020). Simultaneously, networks of forest plot inventories are being complemented by remote‐sensing data, offering novel opportunities to initialize and/or validate model simulation over large spatial scales (Shugart et al., 2015) or complement predictors of SDMs (Fedrigo et al., 2019). Recent advances in remote‐sensing tools, such as the possibility to derive tree‐level information within dense canopies (Ferraz et al., 2016) or fuse spectrometer data with co‐registered LiDAR data (Jucker et al., 2018), provide new ways to parameterize models (e.g., allometries, Fischer et al., 2019; Jucker et al., 2017).

New tools of data processing have been developed to leverage these new sources of data. The development of machine learning techniques offers new possibilities to use the resulting huge datasets for model development and evaluation (Botella et al., 2018; Forkel et al., 2019; Rammer & Seidl, 2019; Reichstein et al., 2019). Additionally, Bayesian and/or inverse modelling approaches can be used to take advantage of diverse sources of data to estimate process parameters, calibrate entire models, and thus reduce model uncertainty (Dietze et al., 2014; Fischer et al., 2019; Hartig et al., 2014; Hartig et al., 2011; LeBauer, Wang, Richter, Davidson, & Dietze, 2013; Lehmann & Huth, 2015; van Oijen et al., 2013; van Oijen et al., 2005). See also Appendix A for more studies that benefit from increasing data availability.

**FIGURE 1:**
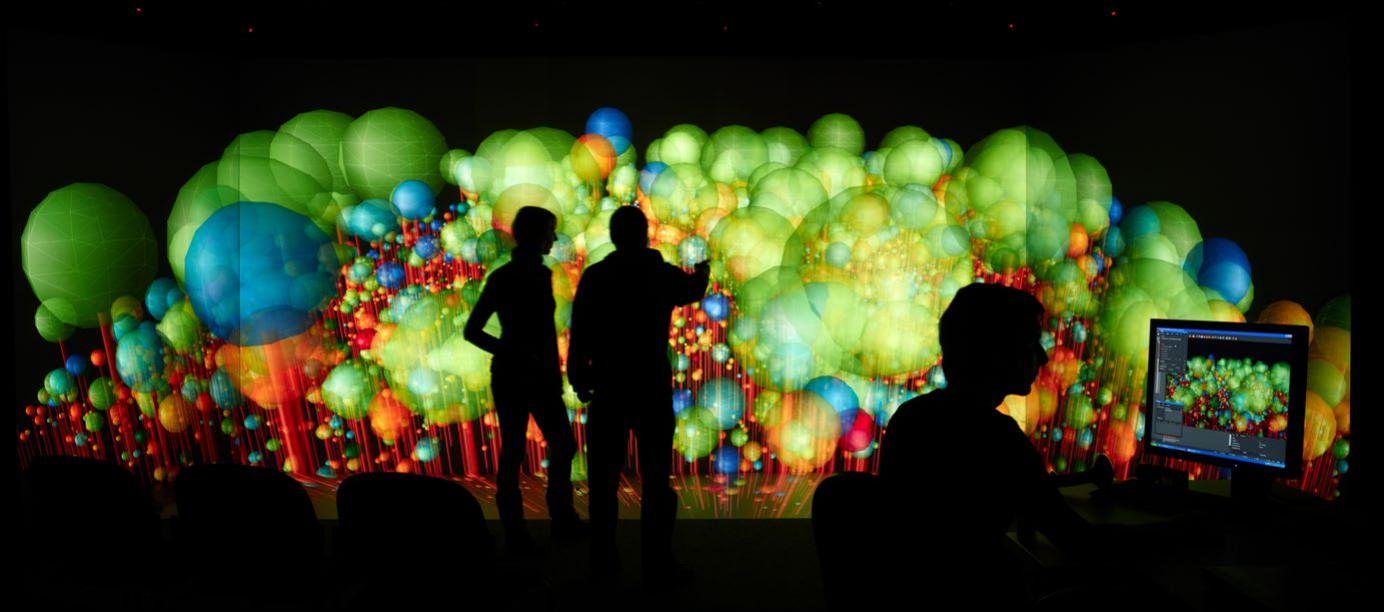
An example of visualization of outputs of a forest model. Visualization of species diversity (crown colors) of a tropical forest simulated by the FORMIND model (Fischer et al., 2016) in the 3D visualization center of UFZ – Helmholtz‐Centre for Environmental Research, Leipzig, Germany

Besides data availability, computing power — in terms of speed and memory — imposes a trade‐off between simulation resolution and coverage, still today limiting large‐scale applications or the fitting of fine‐grained models. For example, the finer‐grained representation of forest biodiversity and structure recently implemented in a DGVM model (LPJmL‐FIT, Sakschewski et al., 2015) was restricted to one biome (Tropics of South America) as opposed to the global scale typically reached by classic DGVM simulations. However, computing power will probably continue to increase in the next years (Kurzweil, 2005), which, together with parallel processing and improved algorithms, allows continuous reduction of computing time (von Bloh et al., 2010; Snell, 2014). As an illustration, using Fast Fourier Transformations for seed dispersal instead of modelling dispersal from each cell to each other increased the computing speed by a factor of 100 (Lehsten et al., 2019). Additionally, remote‐sensing observations allow the up‐scaling of IBMs at lower costs (Rödig et al., 2017, 2018; Shugart et al., 2015). However, a fundamental change of an algorithm in complex models can invoke unplanned side effects, sometimes forcing modelers to invest substantial time and effort to stabilize the new model versions. Furthermore, the development of visualization tools to illustrate simulation results in virtual forest scenes (e.g., Dufour‐Kowalski et al., 2012; Figure 1) represents a valuable lever to communicate on model structure, functioning, and outputs, to inspire for new model developments and applications, but also to detect model errors. See also Appendix B for more examples and details about technical challenges.

### 3.2 Strength in unity: Insights from model inter‐comparison and coupling

Two main types of synergies among models have been increasingly leveraged to better inform forest ecology, namely, model inter‐comparisons and model coupling efforts.

Comparing the outputs from different models that are run under comparable or even identical conditions of driving variables offers valuable insights beyond single‐model simulations. Model comparisons in environmental sciences typically have two main objectives. First, they allow for understanding differences between models by relating the simulated pattern of each model to its underlying processes. This can identify model structural uncertainties, which have been highlighted as a major source of model uncertainties (Famiglietti et al., 2020; Lovenduski & Bonan, 2017; Raiho et al., 2020), and thus foster new model developments as well as novel empirical investigations. Although the increasing complexity of models makes the interpretation of model inter‐comparison results challenging (Fisher & Koven, 2020; Appendix B), model benchmarking is facilitated by new tools of code and data sharing (e.g., Ram, 2013) as well as the availability of detailed standardized databases (Collier et al., 2018; Reyer et al., 2020). Additionally, simulation experiments where different versions of a model are compared allow for insights into the effects of specific process representation in addition to comparisons among models. For example, using 15 different models, including DGVMs and forest gap models, each with alternative mortality sub‐models, Bugmann et al. (2019) explored the influence of different simulated mortality processes on forest dynamics, providing insights into the effects of process uncertainties. The second objective of model inter‐comparison is to provide ensemble simulations that allow for a quantitative assessment of the uncertainties underlying the predictions of the different models.

Model comparisons have a long history within each model community (e.g., among forest gap models — Bugmann et al., 1996 –, forest landscape models — Petter et al., 2020 –, stand‐based eco‐physiological models — Kramer et al., 2002; Morales et al., 2005 –, DGVMs — Cramer et al., 2001; Sitch et al., 2008 –, or SDMs — Araújo & New, 2007). More recently, the increasing ability of different model types to use inputs and provide outputs of similar nature and structure has allowed to compare models across model types (Cheaib et al., 2012), and even across a wide range of sectors such as vegetation, water, agriculture, or biodiversity to study the interaction of these under climate change (Frieler et al., 2017).

Since each model has its own aim, history and therefore specific advantages and limitations (Table 1), the coupling of a model with other types of models can be a valuable approach to expand the initial scope of model applications, or reduce uncertainties in model projections. For instance, several stand‐scale forest models, including IBMs, have been coupled to models of emissions of biogenic volatile organic compounds, revealing that tree species composition and species‐specific emission potentials were important drivers of the feedbacks between climate change and air quality (Keenan et al., 2009a, 2009b; Wang et al., 2018). Similarly, a forest demographic model has been coupled to models of soil microbe‐mediated bio‐geochemistry and competition for nutrients, revealing that spatial variation in soil properties can drive a large variation of forest biomass and composition (Medvigy et al., 2019; see also Sato et al., 2007). SDMs have been coupled to models of habitat colonization in order to take into account dispersal limitation in species distribution projections (Iverson et al., 2004; Nobis & Normand, 2014; see also Franklin, 2010). Fire disturbance models have been implemented in several DGVMs (Lasslop et al., 2014; Schaphoff et al., 2018; Yue et al., 2014, 2015); but also in forest IBMs for a long time (Knapp et al., 2018; Pausas, 1999; Shugart & Noble, 1981), helping to explore different modelling approaches on the interaction between vegetation dynamics and fire (Forkel et al., 2019; Hantson et al., 2016) to explain the declining trend in global burnt area (Andela et al., 2017). More generally, forest models have been coupled to models of disturbances, such as wind storms (Seidl et al., 2011; Thom et al., 2017), allowing the investigation of forest resilience by means of different modeling approaches (Albrich et al., 2020). Other examples include the coupling of a DGVM to a global economy model to dynamically include technical and societal changes in simulating future vegetation dynamics (Dietrich et al., 2019), allowing to investigate the possible trade‐offs between bio‐energy production and several sustainable development goals (Humpenöder et al., 2018).

Model development can also take advantage of the complementarity of different vegetation model types (Table 1) by coupling their different approaches into one model (McMahon et al., 2011). As an illustration, the gap model approach was implemented into a DGVM framework to better account for demographic processes and diversity in regional‐ to continental‐scale studies (e.g., Sakschewski et al., 2015; Smith et al., 2001). Similarly, approaches to include seed dispersal (Lischke et al., 2006), which originate from IBMs (Groeneveld et al., 2009; Urban et al., 1991), can be integrated into large‐scale forest models (Lehsten et al., 2019) to account for dispersal limitation in predictions of species distribution changes under climate change. SDMs have also been coupled to gap models to account for the effects of changes in species distribution at the regional scale on forest composition and functioning at the local scale (García‐Valdés et al., 2020). Model coupling can thus help to improve model realism. In all cases, uncertainties resulting from error propagation across models need to be carefully assessed (e.g., Dunford et al., 2015) to master the resulting increasing complexity while maintaining model reliability and robustness (Famiglietti et al., 2020; Fisher & Koven, 2020; Franklin et al., 2020; Prentice et al., 2015; Saltelli, 2019).

## 4 FOREST MODELLING TO ADDRESS KEY ECOLOGICAL QUESTIONS

Forest models from the different communities have been following converging trajectories of development, leading to a generation of models capable of addressing similar topics and taking on an important role to address novel ecological questions that go far beyond their traditional focus. We identified a number of ecological fields for which we expect forest modelling to make important contributions in the next decade, by increasing our understanding of forest ecosystems and helping generalize ecological findings. To illustrate this, we provide examples of recent model applications to these topics, from the most fundamental to applied ones, and collate ten important questions for future studies (Table 3).

**TABLE 3:** Ten unresolved key questions of forest ecology

We here provide examples of key questions in high need of research effort in forest ecology, for which modelling approaches represent promising tools, as illustrated in the text (see section “Forest modelling to address key ecological questions”). For a more complete list of unanswered ecological questions regarding forest systems, we refer the reader for example, Ammer et al. (2018) or Sutherland et al. (2013).
Q1 How are forest functional and structural characteristics related to climate and soil, and how does this influence forest system functions across space and time?
Q2 Which coexistence mechanisms shape forest communities across environmental gradients?
Q3 How important are rare species for the functioning of forest ecosystems?
Q4 Which forest systems and which of their properties are most sensitive to changes in community composition across scales and why?
Q5 Which factors control the resilience of forest ecosystems to various disturbances?
Q6 What makes forests susceptible to rapid system shifts and how can we project tipping points?
Q7 How do disturbance regimes and global change affect sustainable forest management strategies?
Q8 How do native and invasive tree species move with global change?
Q9 What are the main drivers of carbon allocation within plants and forest ecosystems?
Q10 Why and when do trees die?

### 4.1 Community assembly

Understanding the drivers of community assembly, that is, the processes that shape the number, identity and abundance of co‐occurring species, has been an important question in ecology since its inception (Clements, 1916; Gleason, 1926; MacArthur & Levins, 1967; McGill et al., 2006). Forest models allow for separating the effect of different drivers through the use of null models and sequential simulation set‐ups. For instance, forest IBMs have recently been used to investigate the role of trait‐mediated trade‐offs and their size dependency in shaping forest community (Chauvet et al., 2017; Falster et al., 2017; Kunstler et al., 2009). In doing so, they used a more realistic modelling framework than most theoretical investigations that are generally developed to address these questions and typically restricted to systems with few species. This approach may be further developed and applied to various forest communities as trait data are increasingly becoming available (Box 1). Modelling also helps to disentangle the contribution of stochastic versus deterministic processes through the assessment of variability among repeated runs (Savage et al., 2000).

Although many mechanisms have been identified empirically to contribute to species coexistence in forest communities (Nakashizuka, 2001; Wright, 2002), their relative strengths in observed communities across environmental gradients remain poorly known. Forest modelling could help quantifying their relative contributions through a combination of simple theoretical models and data‐driven simulation experiments, and exploring the debated role of intra‐specific variability on species coexistence (Hart et al., 2016; Lischke & Löffler, 2006; **Q2**, **Q4**, Table 3). To do so, models need to include key aspects of community assembly or known coexistence mechanisms, such as regeneration processes (Vacchiano et al., 2018), negative density‐dependence (Lischke & Löffler, 2006; Maréchaux & Chave, 2017), and functional trade‐offs (Sakschewski et al., 2015) in a heterogeneous environment.

### 4.2 Biodiversity and ecosystem functioning

By virtually manipulating the composition of simulated forest communities, forest IBMs have proven useful in exploring the effect of species richness and functional composition on ecosystem properties (e.g., Fischer et al., 2018). Simulations reproduced positive relationships between (species or functional) diversity and productivity or biomass, in agreement with observed patterns (Maréchaux & Chave, 2017; Morin et al.,^2011^, ^2020^), further motivating a finer‐grained representation of diversity in DGVMs. These studies demonstrated how competition for light can induce this positive effect in heterogeneous forests. Going beyond the effect of bulk species richness, Bohn and Huth (2017) showed that this positive effect is stronger if species are well distributed vertically across the forest canopy. García‐Valdés et al. (2018) showed that climate‐change‐driven extinctions of tree species may affect forest productivity or biomass more severely than random extinctions. Schmitt et al. (2019) found that the mechanisms through which biodiversity influences forest functioning depend on the ecosystem state, shifting from the dominance of the complementarity effect in recently disturbed systems to the dominance of the selection effect in old forests, suggesting a way to reconcile contrasting results obtained with snapshots of ecosystem state in empirical studies.

A more detailed model‐based investigation of the effect of tree species diversity and species loss on other forest ecosystem functions (e.g., water and nutrient cycles) should follow in the near future (**Q1, Q3**, Table 3). Another potential field of model exploration considers the influence of species diversity on crown‐ and surface‐fire intensity as recently investigated empirically for the boreal zone (Rogers et al., 2015). Forest models, including flexible‐trait DGVMs (Sakschewski et al., 2015; Scheiter et al., 2013), could further investigate how functional diversity supports forest productivity and carbon storage under climate change, from the local to the biome scale.

### 4.3 Resilience and stability

Forest models can help to disentangle the different mechanisms shaping forest responses to perturbations through virtual experiments that are beyond the reach of empirical approaches (Albrich et al. 2020). Simulations using an individual‐based and trait‐based DGVM showed that higher trait diversity increases the resilience of the Amazon rainforest under future climate (Sakschewski et al., 2016). This positive effect was attributable to ecological sorting, in agreement with results from forest IBMs in temperate (Morin et al., 2018) and tropical (Schmitt et al., 2019) forests. Higher temporal stability of productivity for forests with higher diversity was also attributed to the asynchrony of species responses to small disturbances (Morin et al., 2014). Using a multi‐model analysis, Radchuk et al. (2019) showed that the multiple properties of stability, such as resistance, recovery, or persistence, (Donohue et al., 2013) can vary independently depending on the disturbance type.

However, we still have an insufficient understanding of forest ecosystem stability that involves multiple processes at various spatial and temporal scales (Donohue et al., 2016), and future modelling studies should help disentangling the multiple drivers of forest resilience while paying attention to the elements leading to feedbacks (e.g., the adult – regeneration feedback). This will foster our predictive ability of potential critical transitions (**Q5**, **Q6**, **Q10**, Table 3).

### 4.4 Carbon stocks and fluxes

The quantification of carbon stocks and fluxes has motivated large efforts of data collection (Table 2), including labor‐intensive forest inventories (Brienen et al., 2015; Ploton, Mortier, Barbier, et al., 2020), flux measurements (Falge et al., 2002; Pastorello et al., 2020), or remote‐sensing (Running et al., 2004; Saatchi et al., 2011). Forest models provide a framework to connect empirical data of various nature, and this connection is even more powerful as models feature resolutions that match with a broader range of empirical data, such as individual‐based modelling approaches, including individual‐based DGVMs (Fisher et al., 2018; Rödig et al., 2017; Sakschewski et al., 2015; Smith et al., 2001).

Models have been used to upscale and infer dynamic estimates of forest productivity and biomass (Fischer et al., 2015) using allometries from field measurements (Chave et al., 2005, 2014). Recently, assimilation of remote‐sensing data within forest models has allowed accounting for the heterogeneity in forest structure and land‐use history in those estimates at stand to continental scales (Joetzjer et al., 2017). For example, by using remote‐sensing‐derived measurements of forest height across a gridded map over the Amazonian basin and a locally optimized gap model, it was possible to estimate the forest successional stages of every cell in this area and derive maps of aboveground biomass and productivity of the whole basin (Rödig et al., 2017, 2018). Beyond estimations of carbon stocks and fluxes, forest models can be used to understand the drivers of their spatial variation. For example, through simulation experiments using an IBM, Fyllas et al. (2017) showed that solar radiation and trait variation driven by spatial species turnover explain the decline of forest productivity along a tropical elevation gradient. Similarly, using a forest demographic model, Berzaghi et al. (2019) showed that elephant disturbances enhance carbon stocks in central African forests through their effects on forest structure and composition. Models can also prove useful to create benchmarks against which other methods to estimate carbon stocks and fluxes can be evaluated and improved (e.g., LiDAR, Knapp et al., 2018; eddy‐flux tower, Jung et al., 2009).

Plant respiration, tree mortality, and carbon allocation are key drivers of forest productivity and biomass (Bugmann & Bigler, 2011; Johnson et al., 2016) but remain poorly understood (Hartmann et al., 2018; Holzwarth, Kahl, Bauhus, & Wirth, 2013; Malhi et al., 2015; Merganičová et al., 2019; Collalti & Prentice, 2019; Collalti, Ibrom, et al., 2020), and future modelling studies should seek to foster our understanding of these critical processes for example, through model‐data fusion approaches (**Q9, Q10**, Table 3).

### 4.5 Forest responses to global change

Models represent a key tool to assess forest responses to the interacting factors of future climate change (Bugmann, 2014; García‐Valdés et al., 2020; Medlyn et al., 2011; Sabaté et al., 2002). Simulating the dynamics of vegetation, including forests, under climate change is the main objective of DGVMs and has been the focus of a sustained effort from this modelling community (Alo & Wang, 2008; Cramer et al., 2001; Friend et al., 2014; Jarvis, 1998; Keenan et al., 2008; Mohren et al., 1997). However, stand‐scale models, such as individual‐based gap models, have also been used to explore forest dynamics under climate‐change scenarios (Bugmann & Fischlin, 1996; Collalti et al., 2018; Fischer et al., 2014; Pastor & Post, 1986; Reyer, 2015; Shugart et al., 2018). Such finer‐scale models can further inform the role of forest composition and structure in shaping forest responses to environmental drivers (Bohn et al., 2018; Fyllas et al., 2017). Additionally, SDMs have been used to project species distributions under future climate change (Noce et al., 2017; Thuiller, 2004), although, as mentioned above, their correlative nature has raised criticisms regarding their use for forecasting under no‐present analogues (Table 1). Overall, a variety of models are utilized to simulate forest responses to climate change, allowing comparisons of different approaches and the assessment of model uncertainties (Cheaib et al., 2012), usually showing that process‐based forest models are more conservative than correlative SDMs (Morin & Thuiller, 2009).

Some recent model developments further aim at accounting for other components of global change (Pérez‐Méndez et al., 2016; Pütz et al., 2014), such as the impacts of defaunation or fragmentation on forest dynamics (Dantas de Paula et al., ,,^2015^, ^2018^; Pütz et al., 2011). Calls for a better integration of plant–animal (Berzaghi et al., 2018) and plant–plant interactions, such as the effect of the increasing liana abundance on tree growth and survival (Verbeeck & Kearsley, 2016), should further foster such developments (di Porcia e Brugnera et al., 2019; Pachzelt, Rammig, Higgins, & Hickler, 2013). Another challenge is the representation of tree species dispersal and migration of tree species at large scales (Lehsten et al., 2019; Neilson et al., 2005; Snell et al., 2014; **Q8**, Table 1), in combination with evolutionary processes to account for species adaptive evolution and trait displacement under environmental changes and fragmentation (DeAngelis & Mooij, 2005; McMahon et al., 2011; Scheiter et al., 2013). Moreover, accounting for the adaptive capacity of tree individuals within their lifetime via acclimation and phenotypic plasticity (Duputié et al., 2015; Richter et al., 2012) remains a challenge, as knowledge about these processes remains incomplete. However, optimality principles may provide a promising approach to predict trait variation with environmental conditions (Franklin et al., 2020). To seek additional insights in estimating future forest responses, a number of studies have used forest models to estimate past forest dynamics (Heiri, Bugmann, Tinner, Heiri, & Lischke, 2006; Lischke, 2005; Lischke et al., 2013; Schwörer et al., 2014). Overall, while differences among model predictions remain large (Prentice et al., 2015), these developments, together with model benchmarking and inter‐comparisons, should help to better understand the long‐term effects of multiple interacting factors of global changes on forests (Seidl et al., 2017; **Q4**, **Q5**, **Q10,** Table 3).

### 4.6 Biodiversity conservation

So far, conservation efforts have not been successful to alleviate biodiversity loss across the globe (Butchart et al., 2010), calling for renewed efforts and biodiversity forecasts (Urban et al., 2016). As SDMs can be calibrated for almost all species for which reliable distribution data are available, these models have long been identified as tools for conservation (Araújo et al., 2019; Davis & Zabinski, 1992; Guisan et al., 2013). Predictions of SDMs under climate‐change scenarios could be used to help refine conservation areas (Ferrier, 2002), or predict invasion ranges of introduced species (Broennimann et al., 2007; Thuiller et al., 2005). Although this claim is put forward frequently (Fernandes et al., 2018), case studies reporting applications remain sparse (Mouquet et al., 2015), likely because of the uncertainty in SDM predictions (Barry & Elith, 2006; Dawson et al., 2011; Journé, Barnagaud, Bernard, Crochet, & Morin, 2020).

Mixed predictions carried out jointly with different model types (process‐based or hybrid distribution models, Evans et al., 2015; Morin & Thuiller, 2009) could provide more robust projections for conservation managers (Thom et al., 2017). Such an approach appears especially feasible for tree species, as individual‐ and process‐based models are typically more abundant for forests than for other ecosystems. Therefore, DGVMs and gap models should be increasingly used to address the challenges of biodiversity conservation planning (e.g., Fischer et al., 2016), in complement to the species‐level process‐based models already available (e.g., Chuine & Beaubien, 2001; Keenan et al., 2011; Serra‐Diaz et al., 2013; **Q1, Q4, Q8**, Table 3).

### 4.7 Forest management

Forests provide important ecosystem services, such as timber production, carbon sequestration, recreation and protection against natural hazards, whose persistence or improvement is of high societal relevance (De Groot et al. 2002, MEA 2005). Sustaining these ecosystem services is the focus of forest management (Nabuurs et al., 2017; Yousefpour et al., 2018). Forest IBMs have a long history in helping management planning (Courbaud et al., 2001; Hiltner et al., 2018; Huth & Ditzer, 2001; Huth et al., 2005; Keenan et al., 2008; Mäkelä et al., 2000; Porté & Bartelink, 2002; Pretzsch et al., 2008). As global change is challenging current and future management strategies (Seidl et al., 2014), forest model development has aimed to help design adaptive forest management practices and mitigation strategies under multiple disturbances (Elkin et al., 2013; Fontes et al., 2010; Kunstler et al., 2013; Lafond et al., 2014; Maroschek et al., 2015; Mina et al., 2017; Rasche et al., 2011; Reyer et al., 2015; Seidl et al., 2018). DGVMs have long disregarded the effect of forest management, as their aggregated representation of vegetation structure typically prevents a realistic representation of tree size distribution and density relevant to simulate silvicultural practices (Table 1). However, some DGVMs used a simplified representation of wood extraction to simulate its effect on forest carbon stocks (Zaehle et al., 2006), and recent efforts have led to the development of more explicit forest management modules, inspired by finer‐scale forest gap models as well as forest growth and yield models (Bellassen et al., 2010; Collalti et al., 2018).

The integration of societal and economic dynamics generates new challenges (**Q7,** Table 3), while future applications and communications with forest stakeholders will benefit from developments regarding visualization of results from forest models (Figure 1).

## 5 CONCLUSION

Forests have multiple important roles for the Earth system and human livelihoods. Sound, quantitative knowledge of forest functioning, structure, and diversity are therefore essential, especially in times of global change. However, many scientific questions regarding forest properties and dynamics remain unresolved, ranging from understanding tree community assembly and projecting forest responses to environmental changes, to assessing the management of forest ecosystems. We illustrated how different forest modelling approaches, due to their continuous development, their complementarity, and mutual enrichment, represent an invaluable toolkit to address ecological questions that require a renewed research effort.

The development of forest models crucially benefits from the interactions among scientists from various fields, within and across modelling communities, but also with field ecologists, physiologists, data scientists, computer engineers, remote‐sensing researchers, and a variety of stakeholders. Owing to their long and successful history in integrating data and knowledge from these various sources, the models used to simulate forests have progressively reached maturity and can tackle a broader array of ecological problems. For instance, forest models can disentangle the drivers of community assembly in forest communities, thus complementing theoretical approaches that typically remain limited to simplified systems. Forest models also provide tractable platforms to perform virtual experiments still out of reach of empirical approaches in forest systems that are characterized by slow dynamics and large spatial extents. This notably allows shedding light on the complex links between forest biodiversity, functioning and resilience in the long term. Furthermore, forest models prove essential to understanding the multiple drivers of forest productivity and biomass by combining field and remote‐sensing data across space and time, and, as a result, provide informed quantifications of carbon stocks and fluxes. Last but not least, ongoing global change and the resulting biodiversity crisis as well as changing climate and disturbance regimes crucially increase the demand of informed projections on forest socio‐ecosystems, for which forest models have a long and successful history, while new developments allow for the integration of an increasing number of interacting factors.

We demonstrated that the converging trajectories of the different modelling approaches used to simulate forests provide new opportunities for comparisons among their outputs. This allows for the quantification of simulation uncertainties and the identification of their sources, and hence fosters new model developments as well as empirical investigations. Overall, iterative model‐data fusion approaches and the resulting cycles of simulation‐assessment‐improvement are continuously increasing the scope of model applications while controlling for simulation uncertainties. Forest models will thus keep contributing to a deeper understanding of forest structure and functioning, and they offer promising routes to fill remaining knowledge gaps and to take on future challenges of forest ecology.

## CONFLICT OF INTEREST

The authors state that there is no conflict of interest.

## AUTHORS’ CONTRIBUTIONS

**Maréchaux I., Langerwisch F., and Bohn F. J.** equally performed conceptualization, data curation, formal analysis, investigation, writing‐original draft, and writing‐review and editing. **Huth A.,** supported for conceptualization, project administration (Lead), and writing‐review and editing. **Bugmann H., Morin X., and Seidl R.** supported for conceptualization and writing‐review and editing. **Reyer C.P.O.** supported for conceptualization, funding acquisition (Lead), and writing‐review and editing. **Collalti A., Dantas de Paula, M., Fischer R., Gutsch M., Lexer M.J., Lischke H., Rammig A., Rödig E., Sakschewski B., Taubert F., Thonicke K., and Vacchiano G.** supported for **w**riting‐review and editing.

## Supporting information

Supplementary Material

Supplementary Material

## Data Availability

No new data were collected in the course of this research.
