# Supplementary material for: Tackling unresolved questions in forest ecology: The past and future role of simulation models
Source: Ecol Evol. 2021 Mar 30;11(9):3746–70. doi: 10.1002/ece3.7391 (PMC8093733; doi:10.1002/ece3.7391)
Supplement: Supplementary file 1 — Supplementary Material [file ECE3-11-3746-s002.docx]

Tackling unresolved questions in forest ecology: the past and future role of simulation models

Maréchaux I.*^1^, Langerwisch F.*/**^2,3^, Huth A^4,5,6^., Bugmann H.^7^, Morin X.^8^, Reyer C.P.O.^9^, Seidl R.^10^, Collalti A.^11,12^, Dantas de Paula, M.^13^, Fischer R.^4^, Gutsch M.^9^, Lexer M.J.^14^, Lischke H.^15^, Rammig A.^16^, Rödig E.^4^, Sakschewski B.^9^, Taubert F.^4^, Thonicke K.^9^, Vacchiano G.^17^ & F. J. Bohn*^4^

## Appendix A: Data availability

Forest models are data-demanding across the different steps of model development and application. A robust parameterization of the multiple processes related to plant life cycle and physiology for diverse plant types, species or individuals requires various data across scales, from plant organ to population, including environmental factors. For many processes, such data are often not available in the required quality and resolution, e.g. for tolerance of trees to resource limitations (Craine, Engelbrecht, Lusk, McDowell, & Poorter 2012; De Kauwe et al., 2015; McMahon et al., 2011) or soil characteristics (Marthews et al., 2014). Additionally, a thorough initialization and validation of forest simulations over large spatial and temporal scales requires observation data encompassing both fine resolution and large coverage over long time spans, which can still be a challenge (Estes et al., 2018, Table 1).

Fortunately, data availability (see Table 2) fosters a systematic model trait-based parameterization for a range of plant species and individuals. For example, Scheiter et al. (2013) and Sakschewski et al. (2015) used reported trait coordination to constrain individual trait combinations in simulations of forest dynamics with DGVMs. In doing so, they improved model representation of functional diversity from a few discrete plant functional types to a continuum of traits, while excluding unrealistic trait combinations (Van Bodegom et al., 2012). Similarly, by taking advantage of comprehensive trait databases, but also of long term inventories and of the detailed information they provide on tree life-histories, forest IBMs have been allowed to simulate hundreds of species within diverse forest communities (Maréchaux & Chave, 2017; Rüger et al., 2019).

Beside networks of forest plot inventories and remote sensing toolscitizen science programs have also been developed to create new opportunities of forest data sampling over large areas (Affouard, Goëau, Bonnet, Lombardo, & Joly 2017; Delbart, Beaubien, Kergoat, & Le Toan 2015; Giraud, Calenge, Coron, & Julliard, 2016; Wäldchen, Rzanny, Seeland, & Mäder, 2018).

The development of machine learning techniques allowed Rammer & Seidl, (2019), to use deep neural networks to estimate vegetation transitions across large spatial scales. Additionally, an example for Bayesian modelling approaches is Van Oijen et al. (2013), who found a strong reduction of uncertainty in most forest models after a Bayesian calibration.

**References**

[Affouard, A., Goëau, H., Bonnet, P., Lombardo, J.-C. & Joly, A. (2017). Pl@ntNet app in the era of deep learning. In: *ICLR 2017 - Workshop Track - 5th International Conference on Learning Representations*. Toulon, France, pp. 1–6.](https://www.zotero.org/google-docs/?a7kVsu)

[Craine, J.M., Engelbrecht, B.M.J., Lusk, C.H., McDowell, N.G. & Poorter, H. (2012). Resource limitation, tolerance, and the future of ecological plant classification. *Frontiers in Plant Science*, 3.](https://www.zotero.org/google-docs/?a7kVsu)

[De Kauwe, M.G., Zhou, S.-X., Medlyn, B.E., Pitman, A.J., Wang, Y.-P., Duursma, R.A.,](https://www.zotero.org/google-docs/?a7kVsu) … Norby, R. J. [(2015). Do land surface models need to include differential plant species responses to drought? Examining model predictions across a mesic-xeric gradient in Europe. *Biogeosciences*, 12, 7503–7518.](https://www.zotero.org/google-docs/?a7kVsu)

[Delbart, N., Beaubien, E., Kergoat, L. & Le Toan, T. (2015). Comparing land surface phenology with leafing and flowering observations from the PlantWatch citizen network. *Remote Sensing of Environment*, 160, 273–280.](https://www.zotero.org/google-docs/?a7kVsu)

[Giraud, C., Calenge, C., Coron, C. & Julliard, R. (2016). Capitalizing on opportunistic data for monitoring relative abundances of species. *Biometrics*, 72, 649–658.](https://www.zotero.org/google-docs/?a7kVsu)

[Estes, L., Elsen, P.R., Treuer, T., Ahmed, L., Caylor, K., Chang, J.,](https://www.zotero.org/google-docs/?a7kVsu) … Ellis, E.C.  [(2018). The spatial and temporal domains of modern ecology. *Nature Ecology & Evolution*, 1.](https://www.zotero.org/google-docs/?a7kVsu)

[Maréchaux, I. & Chave, J. (2017). An individual-based forest model to jointly simulate carbon and tree diversity in Amazonia: description and applications. *Ecol Monogr*](https://www.zotero.org/google-docs/?a7kVsu) *87*(4), 632–664. doi: 10.1002/ecm.1271

[Marthews, T.R., Quesada, C.A., Galbraith, D.R., Malhi, Y., Mullins, C.E., Hodnett, M.G.,](https://www.zotero.org/google-docs/?a7kVsu) … Dharssi, I. [(2014). High-resolution hydraulic parameter maps for surface soils in tropical South America. *Geoscientific Model Development*, 7, 711.](https://www.zotero.org/google-docs/?a7kVsu)

McMahon, S. M., Harrison, S. P., Armbruster, W. S., Bartlein, P. J., Beale, C. M., Edwards, M. E., … Prentice, I. C. (2011). Improving assessment and modelling of climate change impacts on global terrestrial biodiversity. *Trends in Ecology & Evolution*, *26*(5), 249–259. doi: 10.1016/j.tree.2011.02.012

Rammer, W., & Seidl, R. (2019). A scalable model of vegetation transitions using deep neural networks. *Methods in Ecology and Evolution*, *10*(6), 879–890. doi: 10.1111/2041-210X.13171

Rüger, N., Condit, R., Dent, D. H., DeWalt, S. J., Hubbell, S. P., Lichstein, J. W., … Farrior, C. E. (2019). Demographic tradeoffs predict tropical forest dynamics. *BioRxiv*, 808865. doi: 10.1101/808865

[Sakschewski, B., von Bloh, W., Boit, A., Rammig, A., Kattge, J., Poorter, L.,](https://www.zotero.org/google-docs/?a7kVsu) … Thonicke, K.  [(2015). Leaf and stem economics spectra drive diversity of functional plant traits in a dynamic global vegetation model. *Glob Change Biol*, 21, 2711–2725.](https://www.zotero.org/google-docs/?a7kVsu)

[Scheiter, S., Langan, L. & Higgins, S.I. (2013). Next-generation dynamic global vegetation models: learning from community ecology. *New Phytol*](https://www.zotero.org/google-docs/?a7kVsu)

[Van Bodegom, P.M., Douma, J.C., Witte, J.P.M., Ordoñez, J.C., Bartholomeus, R.P. & Aerts, R. (2012). Going beyond limitations of plant functional types when predicting global ecosystem–atmosphere fluxes: exploring the merits of traits-based approaches. *Global Ecology and Biogeography*, 21, 625–636.](https://www.zotero.org/google-docs/?a7kVsu)

[Van Oijen, M., Reyer, C., Bohn, F.J., Cameron, D.R., Deckmyn, G., Flechsig, M., … Rammer, W*.*(2013). Bayesian calibration, comparison and averaging of six forest models, using data from Scots pine stands across Europe. *Forest Ecology and Management*, 289, 255–268.](https://www.zotero.org/google-docs/?a7kVsu)

[Wäldchen, J., Rzanny, M., Seeland, M. & Mäder, P. (2018). Automated plant species identification—Trends and future directions. *PLOS Computational Biology*, 14, e1005993.](https://www.zotero.org/google-docs/?a7kVsu)
