# Supplementary material for: Tackling unresolved questions in forest ecology: The past and future role of simulation models
Source: Ecol Evol. 2021 Mar 30;11(9):3746–70. doi: 10.1002/ece3.7391 (PMC8093733; doi:10.1002/ece3.7391)
Supplement: Supplementary file 2 — Supplementary Material [file ECE3-11-3746-s001.docx]

Tackling unresolved questions in forest ecology: the past and future role of simulation models

Maréchaux I.*^1^, Langerwisch F.*/**^2,3^, Huth A^4,5,6^., Bugmann H.^7^, Morin X.^8^, Reyer C.P.O.^9^, Seidl R.^10^, Collalti A.^11,12^, Dantas de Paula, M.^13^, Fischer R.^4^, Gutsch M.^9^, Lexer M.J.^14^, Lischke H.^15^, Rammig A.^16^, Rödig E.^4^, Sakschewski B.^9^, Taubert F.^4^, Thonicke K.^9^, Vacchiano G.^17^ & F. J. Bohn*^4^

## Appendix B Technical challenges

Several technical obstacles constrain model developments and runtime. Expanding model development and applications relies on code and data sharing within and among larger communities of model developers and users, which is also accompanied by technical challenges. Several modeller teams make the model code (partly) freely available. Additionally, version control systems allow to track changes and collaborate on model code in an efficient way (e.g. Git, Ram, 2013; e.g. Collalti, et al., 2016). Besides code sharing, simulation data are increasingly available following data open access requirements, allowing subsequent analyses or model comparisons. In many modelling studies, the preparation of data (e.g. for input/initialization, calibration or validation) and the analyses of model outputs are very work- and time-intensive. Sharing scripts for analysing forest simulations, e.g. through dedicated platforms (e.g. LeBauer, Wang, Richter, Davidson, & Dietze, 2013) or R (R Core Team, 2018) package (e.g. Duursma, & Medlyn 2012), is also of great help.

Another example of technical challenges is the development of the simulation framework within model intercomparisons and the standardisation of both model inputs and outputs. Moreover, when complex process-based models are involved, whose uncertainties can not simply be attributed to individual processes, a major challenge is to interpret the ensemble runs and to understand which model processes actually explain the differences between models. To address all these issues, transparent model documentations and intensive exchange between modellers is needed accompanied by systematic tests of models and their components (Reyer et al., 2020).

Model coupling is likewise challenging, since, in most models, some processes are hidden in parameters or strongly simplified functions and the model is usually balanced by fitting these parameters. If the simplified process or the parameter is replaced by a more complex sub-model for the process, often the balance can be lost. Additionally, error propagation among models can also prove difficult (Dunford, Harrison, & Rounsevell, 2015). Several model systems and software frameworks have been developed to facilitate multi-model coupling in a systematic way, and they even allow for switching between different models during a simulation (Haas et al., 2013).

# **References**

Collalti, A., Marconi, S., Ibrom, A., Trotta, C., Anav, A., D’Andrea, E., … Santini, M. (2016) Validation of 3D-CMCC Forest Ecosystem Model (v.5.1) against eddy covariance data for 10 European forest sites. *Geoscientific Model Development* 9:479–504.

[Dunford, R., Harrison, P.A. & Rounsevell, M.D.A. (2015). Exploring scenario and model uncertainty in cross-sectoral integrated assessment approaches to climate change impacts. *Climatic Change*, 132, 417–432.](https://www.zotero.org/google-docs/?a7kVsu)

[Duursma, R.A. & Medlyn, B.E. (2012). MAESPA: a model to study interactions between water limitation, environmental drivers and vegetation function at tree and stand levels, with an example application to [CO2]x drought interactions.](https://www.zotero.org/google-docs/?a7kVsu)

[Haas, E., Klatt, S., Fröhlich, A., Kraft, P., Werner, C., Kiese, R., *...*](https://www.zotero.org/google-docs/?a7kVsu) Butterbach-Bahl, K. [(2013). LandscapeDNDC: a process model for simulation of biosphere–atmosphere–hydrosphere exchange processes at site and regional scale. *Landscape Ecol*, 28, 615–636.](https://www.zotero.org/google-docs/?a7kVsu)

[LeBauer, D.S., Wang, D., Richter, K.T., Davidson, C.C. & Dietze, M.C. (2013). Facilitating feedbacks between field measurements and ecosystem models. *Ecological Monographs*, 83, 133–154.](https://www.zotero.org/google-docs/?a7kVsu)

[R Core Team. (2018). *R: a language and environment for statistical computing*. R Foundation for Statistical Computing, Vienna, Austria.](https://www.zotero.org/google-docs/?a7kVsu)

[Ram, K. (2013). Git can facilitate greater reproducibility and increased transparency in science. *Source Code for Biology and Medicine*, 8, 7.](https://www.zotero.org/google-docs/?a7kVsu)

Reyer, C., Silveyra Gonzalez, R., & Dolos, K. (2020). The PROFOUND database for evaluating vegetation models and simulating climate impacts on forests. *(in press)*

# 
